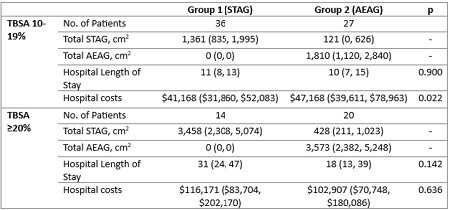# 83 Outcomes Using Autologous Epidermal Autograft for Wound Closure of Majority Deep Partial Thickness Burns

**DOI:** 10.1093/jbcr/irae036.082

**Published:** 2024-04-17

**Authors:** Andrea Abeln, James Klugh, Deepanjli Donthula, Catherine Almand, Taylor Campbell, Chuantao Jiang, Daniel J Freet, Todd F Huzar, David J Wainwright, Charles E Wade, Lillian S Kao, John A Harvin

**Affiliations:** McGovern Medical School at UTHealth Houston, Houston, TX; McGovern Medical School at UTHealth Houston, Houston, TX; McGovern Medical School at UTHealth Houston, Houston, TX; McGovern Medical School at UTHealth Houston, Houston, TX; McGovern Medical School at UTHealth Houston, Houston, TX; McGovern Medical School at UTHealth Houston, Houston, TX; McGovern Medical School at UTHealth Houston, Houston, TX; McGovern Medical School at UTHealth Houston, Houston, TX; McGovern Medical School at UTHealth Houston, Houston, TX; McGovern Medical School at UTHealth Houston, Houston, TX; McGovern Medical School at UTHealth Houston, Houston, TX; McGovern Medical School at UTHealth Houston, Houston, TX

## Abstract

**Introduction:**

Autologous epidermal autografting (AEAG) closes wounds with a significantly smaller donor site compared with traditional split thickness autograft (STAG). Theoretically, treatment of large burns with a smaller required donor site may reduce length of stay (LOS) and overall costs. We hypothesized that AEAG would be associated with reduced overall hospital costs in patients with ≥10% total body surface area (TBSA), majority deep partial thickness burns compared to STAG alone.

**Methods:**

Burn center patients from 2018-2023 with ≥10% TBSA, operative, majority deep partial thickness burns were split into two groups based on treatment strategy: Group 1 – patients who underwent STAG only versus Group 2 - patients who underwent AEAG with STAG reserved only for full thickness areas. Primary outcomes included length of stay and overall costs. Groups were stratified by percent TBSA (10-19% and ≥20%). Univariate and multivariate analyses were performed.

**Results:**

The entire cohort included 97 patients, 50 (52%) in Group 1 and 47 (48%) in Group 2. The majority of the cohort (n=63, 65%) had 10-19% TBSA burns and 34 (35%) had ≥20% TBSA burns. In the 10-19% stratum, there were no differences in demographics or injury characteristics. Groups 1 and 2 had similar lengths of stay but Group 2 had higher overall costs. In the ≥20% TBSA stratum, there were no differences in demographics or injury characteristics except that Group 2 had a higher proportion of male patients. In the 10-19% TBSA stratum, after adjusting for age and TBSA, Group 2 was associated with increased costs (RR 1.46, 95% CI 1.18–1.80, p=0.001) and LOS (RR 1.16, 95% CI 1.00–1.34, p=0.045). In the ≥20% TBSA stratum and after adjusting for age and TBSA, Group 2 was associated with no difference in cost (RR 0.89, 95% CI 0.45 – 1.69, p=0.717) and a reduction in LOS (RR 0.78, 95% CI 0.68–0.88, p< 0.001).

**Conclusions:**

For ≥10% TBSA, majority deep partial thickness burns, the use of AEAG with STAG reserved only for full thickness areas was associated with a reduction in the need for STAG. There is a potential for reduced length of stay without increased costs in burns ≥20% TBSA with AEAG use. However, a larger, adequately powered trial is necessary to validate this finding.

**Applicability of Research to Practice:**

AEAG was associated with a reduction in overall STAG requirement in all patients and was not associated with an increased cost for large % TBSA majority deep partial thickness burns.